# Let them eat virus: exploring how TBK1 (TANK binding kinase 1) enhances autophagic flux to promote autophagic degradation of coxsackievirus B

**DOI:** 10.1080/27694127.2022.2139332

**Published:** 2022-10-31

**Authors:** Savannah Sawaged, Jon Sin

**Affiliations:** a The Smidt Heart Institute, Cedars-Sinai Medical Center, Los Angeles, California, USA; b Department of Biological Sciences, University of Alabama, Tuscaloosa, Alabama, USA

**Keywords:** Antiviral immunity, autophagy, coxsackievirus, pancreatitis, vesicles, viral egress, virus

## Abstract

Coxsackievirus B (CVB) is a common human enterovirus that can cause an array of systemic inflammatory diseases. We and others have demonstrated that macroautophagy/autophagy is activated during CVB infection leading to viral engulfment within autophagosomes. Interestingly, rather than this mechanism leading to bulk degradation of intracellular virus (also referred to as xenophagy), autophagosome-lysosomal fusion appears to be circumvented, leading to extracellular release of CVB via ejected autophagosomes. In our present study, we have found that TBK1 (TANK binding kinase 1) plays a role in limiting CVB infection by promoting autophagic flux to limit autophagy-based viral egress. This aspect of viral defense also appears to be independent of TBK1’s well-characterized involvement in interferon signaling. Indeed, genetic inhibition of *TBK1* significantly enhances CVB infection *in vitro* and dramatically increases the amount of vesicle-bound virus being released from the cell. Furthermore, inhibition of TBK1 via amlexanox treatment markedly increases serum levels of infectious extracellular vesicles (EV) and severity of pancreatitis in CVB-infected mice. In all, the identification of TBK1’s involvement in the suppression of CVB egress pegs it as a promising therapeutic target for the development of novel antiviral strategies against not only CVB but potentially other viruses that exploit autophagy to promote viral spread.

The enterovirus coxsackievirus B (CVB) is a ubiquitous human pathogen that is common in both first-world and developing countries. Though CVB infections are typically subclinical or may cause self-resolving flu-like symptoms, severe CVB infections can manifest a variety of systemic illnesses such as meningo-encephalitis, myocarditis, and pancreatitis. Young children and infants (<2 years) are particularly susceptible to severe CVB diseases with higher mortality, but these diseases can occur in adults as well. Additionally, juvenile CVB illnesses can sometimes become chronic and present severe sequelae long after infection. As with all enteroviruses, CVB is a non-enveloped virus; being thought to predominantly rely on cytolysis to promote viral egress. We and others, however, had demonstrated that CVB and other enteroviruses induce cellular autophagy, become engulfed in autophagosomes, and become expelled from the cell in infectious virus-laden vesicles. Not only does this mode of viral release prolong host cell life and therefore viral replication, but individual vesicular structures often can also house numerous viral particles, and it is speculated that these membranes can mask the virus from neutralizing antibodies. Additionally, we had previously demonstrated that these vesicles are also enriched with proviral microRNA which could further enhance subsequent waves of infection.

In order for virus-laden autophagosomes to become released from the cell, CVB would presumably have to circumvent autophagic flux in order to evade lysosomal degradation. In the current study, we have demonstrated that TBK1 (TANK binding kinase 1) is involved in promoting autophagic flux and degradation of intracellular CVB [1]. Indeed, we found that inhibition of TBK1 not only results in significant elevations in vesicle-based CVB egress and increases in overall infection, in a mouse model of viral pancreatitis, blocking TBK1 also exacerbates pancreatic destruction and leads to a rise in pancreatic and serum viral titers. This aspect of TBK1’s viral effects appear to be independent of TBK1’s well-known involvement in interferon signaling. Conversely, activation of TBK1 *in vitro* with manassantin B inhibits viral replication and spread. We found that manassantin B treatment boosts basal levels of autophagic flux and prevents excessive autophagosome accumulation when flux is blocked. TBK1 activation hampers the extracellular release of infectious virus and viral protein by efficient trafficking of virus-containing autophagosomes to their degradative fate (Figure 1). Future studies using manassantin B to treat viral pancreatitis might provide a new tool favoring autophagy-mediated antiviral mechanisms and open new avenues for therapeutic purposes.

**Figure 1.**
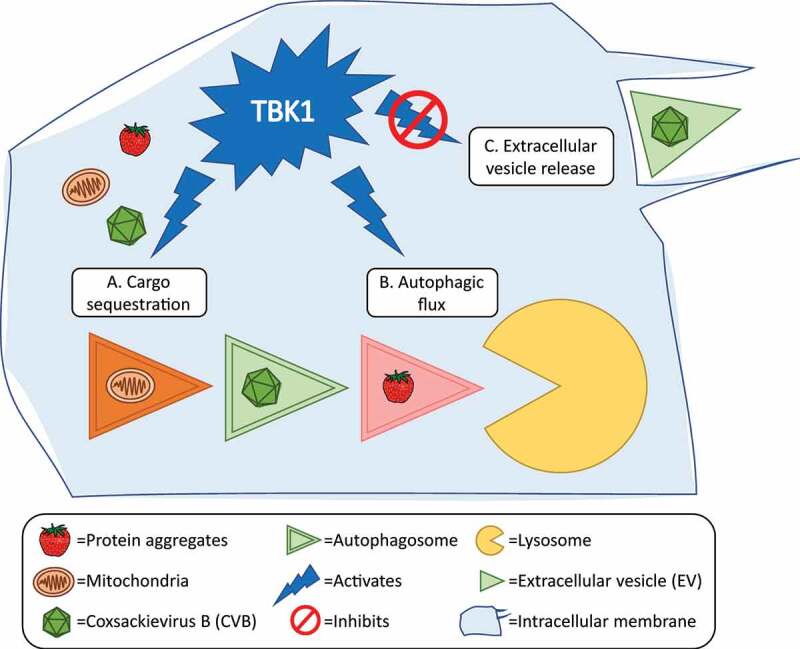
Schematic of TBK1 function in antiviral autophagy. (**A**) Upon infection, CVB localize to mitochondria and trigger mitochondrial fission. Autophagy is initiated and TBK1 activates autophagy receptors for efficient cargo sequestration (mitochondrial fragments, CVB, protein aggregates) within autophagosomes. (**B**) TBK1 activates mitochondrial receptors to ensure autophagosome fusion to the lysosome and eventual cargo degradation and to (**C**) prevent the non-lytic release of CVB-laden extracellular vesicles into the extracellular space.

Furthermore, we characterized and ascribed a novel function in autophagy modifiers during CVB infection. This was demonstrated by several *in vitro* experiments using siRNA-mediated knockdown of *GABARAPL1* and *GABARAPL2*, both downstream targets of TBK1 phosphorylation. These GABARAP paralogs are trafficking proteins that are important for phagophore expansion and autophagosome fusion with the lysosome to complete autophagy. During normal autophagy, TBK1 phosphorylation of Atg8-family member proteins such as MAP1LC3C and GABARAPL2 prevent their premature removal from the autophagosome and ensure fusion with the lysosome. Therefore, we hypothesized that *TBK1* knockdown enhances infectious EV release due to disruption in GABARAPL1 and GABARAPL2-mediated autophagic flux. Dual knockdown of *GABARAPL1* and *GABARAPL2* inhibits intracellular CVB infection and infectious EV release substantially, thus indicating that GABARAPL1 and GABARAPL2 exert important roles in coordinating viral elimination.

The clinical manifestations of autophagy dysregulation and *TBK1* deficiency are documented in central nervous system (CNS) disorders. Protein aggregation of TARDBP/TDP-43 is a hallmark of amyotrophic lateral sclerosis (ALS) and frontotemporal dementia (FTD) pathology which has specifically been described in motor neurons harboring *TBK1* mutations. Further, CVB causes proteinopathy and aggregation of TARDBP, which prevents the biological activity of TARDBP in alternative RNA splicing. There are multiple *TBK1* mutations that have been described in ALS and FTD which warrant studies to understand if CVB is a causative agent in adult CNS disorders. Our group had previously demonstrated the ability of CVB to target the CNS and infect neural stem cells in neonatal mice, leading to long-term developmental defects and neurological sequelae. Antiviral treatment partially reverses some of the developmental defects administered 30 days post-infection. Taken together, novel therapies targeting autophagy or mitophagy could benefit patients experiencing (1) virus-related pathology long after active infection or (2) autophagy-related pathologies with unknown etiology.

In all, our recent report highlights the valuable role that xenophagic degradation of CVB plays in cellular antiviral defense. TBK1 appears to play a central role in mediating viral degradation, and, given TBK1’s additional yet seemingly independent role in antiviral cytokine production, TBK1 seems to govern protection over a wide range of viruses, even those that are classically adept at disrupting interferon responses. This further positions TBK1 as a valuable therapeutic target for the development of novel antivirals.
